# Clinical Features and Outcomes of *Myroides* Species Infections

**DOI:** 10.1093/ofid/ofaf049

**Published:** 2025-01-28

**Authors:** Mayyadah H Alabdely, Kristin Englund, Nabin K Shrestha

**Affiliations:** Infectious Disease Department, Cleveland Clinic, Cleveland, Ohio, USA; Infectious Disease Department, Cleveland Clinic, Cleveland, Ohio, USA; Infectious Disease Department, Cleveland Clinic, Cleveland, Ohio, USA

**Keywords:** antibiotic resistance, cohort study, *Myroides species*, outcomes, survival

## Abstract

**Background:**

*Myroides* species, Gram-negative bacilli from the *Flavobacteriaceae* family, are typically considered low-virulence pathogens but have previously been described as extensively drug-resistant. This study investigates the clinical features and outcomes of *Myroides* infections.

**Methods:**

We conducted a retrospective cohort study of patients hospitalized at Cleveland Clinic with *Myroides* infection. Infections were defined as a positive *Myroides* culture from a sterile site that were treated with an antibiotic to which the isolate was susceptible. Controls were hospitalized patients with a positive culture for *Myroides*, who did not meet the definition for infection. Survival for infected patients and uninfected controls was compared using Cox proportional hazards regression.

**Results:**

Between January 2015 and September 2023, 52 positive *Myroides* species cultures were identified, with 21 deemed infections. A higher proportion of *Myroides*-infected patients than controls had diabetes mellitus. The most common infections were skin/soft-tissue infections (42.8%), osteomyelitis (33.3%), and urinary tract infections (19%); and 28.5% were bacteremic infections. All isolates were resistant to aminoglycosides, but the majority were susceptible to trimethoprim-sulfamethoxazole (81%), ciprofloxacin (57%), and meropenem (68%). The main antimicrobial treatments provided were meropenem, ciprofloxacin, and trimethoprim-sulfamethoxazole. A significant difference in survival was not found between patients with *Myroides* infection and uninfected controls (hazard ratio, 3.42; 95% confidence interval, .63–18.74; *P* = .16).

**Conclusions:**

All patients in this study had reasonable treatment options, belying previous reports of extensive antibiotic resistance in *Myroides*. Our small study did not detect a statistically significant decrease in survival among *Myroides*-infected patients compared to controls.

## Teaser

Between 2015 and 2023, 21 clinically significant *Myroides* infections were identified at Cleveland Clinic. Infections included osteomyelitis, urinary tract infections, and skin and soft-tissue infections. Our isolates were all resistant to aminoglycosides. The study did not find significantly lower survival among infected patients compared to colonized but uninfected controls.

Bacteria belonging to the genus *Flavobacterium* were first identified in 1923. Among these, *Flavobacterium odoratum* is an aerobic, yellow-pigmented, nonmotile, and nonfermenting gram-negative rod, notable for its distinct fruity odor. Because of unique characteristics observed in *F odoratum*, a new genus, *Myroides,* was established in 1996. This new genus initially included 2 species: *Myroides odoratus* (formerly *F odoratum) and Myroides odoratimimus* [1].

*Myroides* are widely distributed in nature and are prevalent in water and soil [2]. Two species are commonly associated with disease: *M odoratus* and *M odoratimimus* [2]. The first strains *Flavobacterium odoratum Stutzer* and *Kwaschnina* were originally isolated from the feces of patients suffering from typhoid fever and gastroenteritis [3]. Although generally considered low risk, they do affect immunocompromised individuals, and infections that have previously been attributed to *Myroides* include urinary tract infections (UTIs), skin and soft tissue infections, and osteomyelitis [4–7].

As there have only been case reports and case series published on *Myroides* infection risk factors for *Myroides* infection have not been identified, but associations with prolonged hospitalization, invasive or surgical procedures, diabetes, and chronic kidney disease have been speculated [8]. Additionally, environmental factors such as exposure to animals, unsanitary conditions, or contact with contaminated water or soil have been hypothesized because of the organism's origin as an environmental pathogen [9].

The ability of *Myroides* to form biofilms [10] and their reported intrinsic antimicrobial resistance [3,9] suggest that treating such infections may be challenging. However, published studies on *Myroides* infection are limited to a few case reports and case series [1,4–7].

The purpose of this study was to examine the clinical manifestations, diagnostic methods, susceptibility patterns, treatment strategies, and outcomes of a cohort of patients with *Myroides* infection encountered at a large tertiary care center.

## METHODS

### Study Design

This was a retrospective cohort study conducted at the Cleveland Clinic Health System in the United States from January 2015 to September 2023.

### Patient Consent Statement

The study was approved by the Cleveland Clinic institutional review board as exempt research (IRB #23-1243). A waiver of informed consent and waiver of Health Insurance Portability and Accountability Act authorization were approved to allow the research team access to the required data.

### Pathogen Identification and Susceptibility Testing

All isolates were identified by matrix-assisted laser desorption/ionization time-of-flight mass spectrometry using the VITEK system, comparing protein spectra of isolates to a reference database for precise species-level identification. Identification of *Myroides* was limited to the species level. Antibiotic susceptibility testing was performed using broth microdilution. In describing the proportions susceptible and resistant, the minimum inhibitory concentration results were interpreted in accordance with the Clinical and Laboratory Standards Institute standards (2024) breakpoints for non-*Enterobacteriaceae* [11], as follows:

- Ceftriaxone: S ≤ 1 µg/mL, I = 2 µg/mL, R ≥ 4 µg/mL.
- Cefepime: S ≤ 8 µg/mL, I = 16 µg/mL, R ≥ 32 µg/mL.
- Piperacillin-tazobactam: S ≤ 16/4 µg/mL, I = 32/2–64/2 µg/mL, R ≥ 128/2 µg/mL.
- Meropenem: S ≤ 4 µg/mL, I = 8 µg/mL, R ≥ 16 µg/mL.
- Levofloxacin: S ≤ 2 µg/mL, I = 4 µg/mL, R ≥ 8 µg/mL.
- Ciprofloxacin: S ≤ 1 µg/mL, I = 2 µg/mL, R ≥ 4 µg/mL.
- Trimethoprim-sulfamethoxazole: S ≤ 2/38 µg/mL, I = − µg/mL, R ≥ 4/76 µg/mL.
- Gentamicin: S ≤ 4 µg/mL, I = 8 µg/mL, R ≥ 16 µg/mL.
- Amikacin: S ≤ 16 µg/mL, I = 32 µg/mL, R ≥ 32 µg/mL.
- Tobramycin: S ≤ 4 µg/mL, I = 8 µg/mL, R ≥ 16 µg/mL.

### Identification of Infected Patients and Controls

Patients with cultures positive for *Myroides* were classified into those with clinically significant *Myroides* infection and colonized but uninfected controls. Clinically significant *Myroides* infections were defined as those with isolates from sterile sites (eg, blood, urine, deep tissues) in patients presenting with signs and symptoms of infection and where a decision was made to treat with antibiotics. Patients who had cultures positive for *Myroides* but did not meet these definitions for infection were considered controls.

### Statistical Analysis

Survival up to 90 days was compared between patients with *Myroides* infection and controls via cumulative incidence curves. Risk of death between those with *Myroides* infection and controls was evaluated using Cox proportional hazards regression. Analyses were done using R version 4.4.0 [12].

## RESULTS

Of the 52 patients with cultures positive for *Myroides* during the study period, 21 were classified as *Myroides* infection. The remaining 31 were considered colonized but uninfected controls.

### Description of Patients With *Myroides* Infection and Controls

Of the 21 clinically significant infections identified in *Myroides*-infected patients, 42.8% were skin and soft-tissue infections (three associated with bacteremia), 33.3% were cases of osteomyelitis (including 2 with bacteremia), and 19% were urinary tract infections (none bacteremic). One additional case was diagnosed as bacteremia where the primary focus of infection remained undiagnosed.

The most commonly administered antibiotics were meropenem, ciprofloxacin, and trimethoprim-sulfamethoxazole, each given to 6 patients (28.5%). Four patients developed sepsis, leading to death within 24 hours to 30 days following a positive culture.

A more detailed description of individual cases is provided in Supplementary Table 1.

There were no significant age or sex differences between *Myroides*-infected patients and noninfected controls. However, a higher proportion of the *Myroides*-infected patients had diabetes mellitus (57.1% vs 16.1%, *P* = .005). The mean of the most recent HbA1c level was also higher in *Myroides*-infected diabetic patients (7.24 g/dL) compared to noninfected diabetic controls (6.6 g/dL).

Of the 4 patients with *Myroides* UTI, 2 patients (50%) had benign prostatic hyperplasia (BPH). None of the controls had BPH.

Characteristics of *Myroides*-infected patients and uninfected but colonized controls are compared in Table 1.

**Table 1.** Patient Characteristics Stratified by Myroides Infection Status

Characteristic^a^	No *Myroides* Infection (n = 31)	*Myroides* Infection (n = 21)	*P* Value
Demographics	…	…	
Age, mean (SD)	62.8 (14.3)	64.5 (8.4)	.628
Male sex	28 (90.3)	14 (66.7)	.078
Comorbidities	…	…	
Benign prostatic hyperplasia	0 (0.0)	3 (15.0)	-
Coronary artery disease	1 (3.2)	4 (19.0)	.156
Cancer, any type	2 (6.5)	2 (9.5)	1.000
Diabetes mellitus	5 (16.1)	12 (57.1)	.005
Heart failure	6 (19.4)	6 (28.6)	.661
Hypertension	13 (41.9)	10 (47.6)	.904
Liver disease	1 (3.2)	2 (15.4)	.421
Peripheral arterial disease	5 (16.1)	6 (28.6)	.464
HIV positive	0 (0.0)	1 (4.8)	-
Chronic kidney disease	4 (12.9)	8 (38.1)	.075

Abbreviation: SD, standard deviation.

^a^Represented as n (%) unless specifically mentioned otherwise.

### Antimicrobial Resistance

Antimicrobial susceptibility testing of isolates from the 21 infected patients showed that all isolates were resistant to aminoglycosides, with limited susceptibility to piperacillin-tazobactam (2 isolates). Thirteen (68%) of 19 isolates were susceptible to meropenem. The majority of isolates were susceptible to trimethoprim-sulfamethoxazole (81%) and ciprofloxacin (57%).

Antimicrobial susceptibility profiles of the isolates from infected patients are summarized in Table 2.

**Table 2.** Antibiotic Susceptibilities for Isolates Tested

Antibiotic	Number Tested	Susceptible N (%)	Intermediate N (%)	Resistant N (%)
Piperacillin-tazobactam	20	2 (10)	15 (75)	3 (15)
Cefepime	15	4 (26.6)	6 (40)	5 (33)
Ciprofloxacin	21	12 (57.14)	5 (23.8)	4 (19)
Levofloxacin	4	3 (75)	0	1 (25)
Amikacin	18	0	0	18 (100)
Gentamicin	21	0	0	21 (100)
Tobramycin	20	0	0	20 (100)
Meropenem	19	13 (68.4)	5 (26.3)	1 (5.2)
Sulfamethoxazole-trimethoprim	21	17 (80.9)	0	4 (19)

Two of the isolates were resistant to ciprofloxacin and trimethoprim-sulfamethoxazole so oral treatment options were limited.

### Survival of Patients With *Myroides* Infection

**Figure 1.**
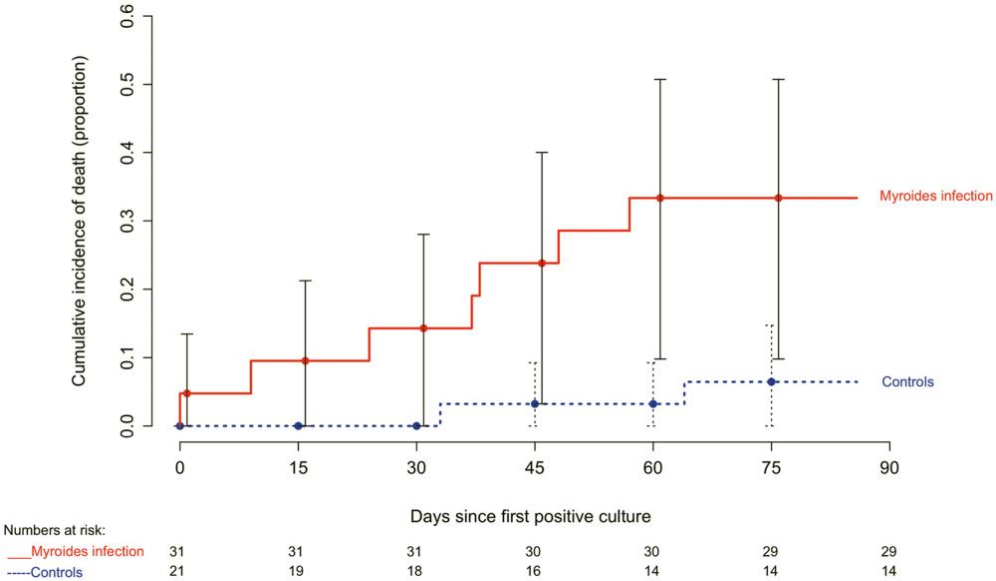
Cumulative incidence of death for patients with *Myroides* infection and controls.

Figure 1 compares survival among patients with *Myroides* infection (all of whom received directed antimicrobial therapy) and colonized but uninfected controls (none of whom were treated with antibiotics directed against *Myroides*). This study was unable to find a significant difference in survival up to 90 days between patients with treated *Myroides* infection and colonized but uninfected controls (hazard ratio, 3.42; 95% confidence interval, .63–18.74; *P* = .16).

## DISCUSSION

This study is the largest cohort study of confirmed *Myroides* cases. It found that the most common infections caused by *Myroides* spp. were skin/soft-tissue infections, osteomyelitis, and UTIs. Unlike the characterization of this pathogen as being extensively drug-resistant in prior case reports and case series [3,7,9,13], our study did not find *Myroides* to be extensively drug-resistant. In a study conducted by Gunzer et al. [14], 59 strains of *Myroides* species were tested for resistance against 20 commonly used antibiotics. Most of the strains in that study were resistant to ciprofloxacin in contrast to our study findings where the majority of isolates (57.14%) were susceptible. Gunzer et al. also reported that their strains appeared naturally resistant to the aminoglycosides gentamicin and amikacin, which aligns with our findings, as all isolates in our study were resistant to all aminoglycosides tested. Additionally, most isolates in both our study and the study by Gunzer et al. were susceptible to meropenem.

Comparing outcomes between infected patients and colonized but uninfected controls allowed for evaluating outcomes attributable to *Myroides* infection. The most important limitation of this study is its small sample size. Although our study was unable to find a statistically significant difference in survival between *Myroides*-infected patients and uninfected controls, almost one fifth of the *Myroides* infected patients in our study died of sepsis, and it is possible that a mortality risk was missed because of an insufficient sample size. The small sample size also precluded adjustment for confounding factors in evaluation of survival. Although the description of clinical characteristics suggested some interesting findings, such as 50% of patients with *Myroides* UTI having BPH, our study was not designed to identify risk factors for *Myroides* infection.

*Myroides* do not appear to be as extensively drug-resistant as previously portrayed. More research is needed to better understand this pathogen and risk factors for infection. Larger studies are needed to evaluate clinical outcomes following infection.

## Supplementary Material

ofaf049_Supplementary_Data

## Supplementary Data

Supplementary materials are available at *Open Forum Infectious Diseases* online. Consisting of data provided by the authors to benefit the reader, the posted materials are not copyedited and are the sole responsibility of the authors, so questions or comments should be addressed to the corresponding author.

## Notes

***Acknowledgments.*** We thank our microbiology laboratory for their work on identification and susceptibility testing.

***Author contributions.*** M.H.A.: Conceptualization, methodology, investigation, data curation, writing–original draft preparation. K.E.: Project administration, resources, writing–reviewing and editing. N.K.S.: Methodology, validation, software, formal analysis, visualization, writing–reviewing and editing, supervision.

***Financial support.*** None.
