# Supplementary material for: Clinical Features and Outcomes of Myroides Species Infections
Source: Open Forum Infect Dis. 2025 Jan 28;12(2):ofaf049. doi: 10.1093/ofid/ofaf049 (PMC11832043; doi:10.1093/ofid/ofaf049)
Supplement: ofaf049_Supplementary_Data [file ofaf049_supplementary_data.docx]

Table 1: Summary of the 21 cases of *Myroides* infection

|  | Age /Gender | Co-morbidities / predisposing condition | Type of infection | Bacteremia | Surgically debrided | Antibiotic used | Outcome at 90 D |
| --- | --- | --- | --- | --- | --- | --- | --- |
| 1 | 63/Male | DM, afib, HTN | Skin & soft tissue | No | Yes | MER, CTX | Survived |
| 2 | 66/Male | PAD, DM, HTN, CKD4 | OM | No | Yes | CIP, CLD | Survived |
| 3 | 64/Male | PAD, HTN, CAD, afib, CKD3, HF | OM | No | Yes | CIP, MTZ | Survived |
| 4 | 68/Male | CKD4 ,BPH | UTI | No | NA | TMP/SXT | Survived |
| 5 | 69/Male | BPH, Urine retention, CKD4 | UTI | No | NA | TMP/SXT | Survived |
| 6 | 62/Male | PAD, DM, HTN | Skin& soft tissue | No | Yes | TZP | Survived |
| 7 | 66/Female | DM,HF,HTN,CKD3 | Skin& soft tissue | No | No | CIP, TMP/SXT | Survived |
| 8 | 64/Male | DM, HF, CKD5, HIV | Skin & soft tissue infection of LL with bacteremia | Yes | No | MER | Survived |
| 9 | 63/Male | DM, HF, HTN, CAD, BPH,CKD3 | Skin& soft tissue | No | No | MER, TZP | Died  within 30 days |
| 10 | 73/Female | PAD, HTN | OM | No | Yes | VAN, TZP, CIP | Died  within 9 days |
| 11 | 58/Male | DM | Skin& soft tissue | No | No | ERT, TMP/SXT | Survived |
| 12 | 88/Female | DM, afib, HF, HTN | OM with bacteremia | Yes | No | MER | Died 27 days |
| 13 | 55/Female | Cervical cancer, hemoorhagic cystitis | UTI | No | No | TMP/SXT | Survived |
| 14 | 63/Female | cervical cancer, DM,CKD4 | UTI | No | No | CIP | Survived |
| 15 | 44/Male | DM,CAD, dog chewed on his toe | OM | No | Yes | AMX/CLV, TMP/SXT | Survived |
| 16 | 58/Male | PAD, HTN | OM | No | Yes | CEP | Survived |
| 17 | 70/Male | PAD, DM, CAD, HTN | Skin& soft tissue | No | Yes | MER, VAN | Survived |
| 18 | 58/Male | None | Bacteremia (unclear source) | Yes | No | none | Died  within less than 24 hours |
| 19 | 72/Male | Liver cirrhosis | Skin & soft tissue infection with Bacteremia | Yes | No | CIP | Survived |
| 20 | 65/Female | Liver cirrhosis | Skin & soft tissue infection with Bacteremia | Yes | No | LVX | Survived |
| 21 | 66/Female | Sacral wound covered with maggots | OM with Bacteremia | Yes | Yes | MER | Survived |

Abbreviations: DM, Diabetes mellites; HTN, hypertension; CAD, coronary artery disease; PAD, peripheral artery disease; HF, heart failure; CKD, chronic kidney disease; afib, atrial fibrillation; MIC, minimum inhibitory concentration; R, resistant; I, intermediate; S, sensitive; TZP, piperacillin–tazobactam; CEF, cefepime; CIP, ciprofloxacin; AK, amikacin; GN, gentamicin; MER, meropenem; TMP/SXT, trimethoprim–sulfamethoxazole; LVX, levofloxacin; AUG, augmentin ; ERT, Ertapenem ; CLD, clindamycin; MTZ, metronidazole ​
